# Design of Lightweight Driver-Assistance System for Safe Driving in Electric Vehicles

**DOI:** 10.3390/s19214761

**Published:** 2019-11-02

**Authors:** Shabir Ahmad, Sehrish Malik, Dong-Hwan Park, DoHyeun Kim

**Affiliations:** 1 Department of Computer Engineering, Jeju National University, Jeju 63243, Korea; shabir@jejunu.ac.kr (S.A.); serrym29@gmail.com (S.M.); 2 Electronics and Telecommunications Research Institute, Daejeon 34129, Korea; dhpark@etri.re.kr

**Keywords:** autonomous vehicle, internet of things, real-time systems, input task modeling, embedded devices, mixed-criticality task scheduling

## Abstract

Electric-vehicle technology is an emerging area offering several benefits such as economy due to low running costs. Electric vehicles can also help to significantly reduce CO_2_ emission, which is a vital factor for environmental pollution. Modern vehicles are equipped with driver-assistance systems that facilitate drivers by offloading some of the tasks a driver does while driving. Human beings are prone to errors. Therefore, accidents and fatalities can happen if the driver fails to perform a particular task within the deadline. In electric vehicles, the focus has always been to optimize the power and battery life, and thus, any additional hardware can affect their battery life significantly. In this paper, the design of driver-assistance systems has been introduced to automate and assist in some of the vital tasks, such as a braking system, in an optimized manner. We revamp the idea of the traditional driver-assistance system and propose a generic lightweight system based on the leading factors and their impact on accidents. We model tasks for these factors and simulate a low-cost driver-assistance system in a real-time context, where these scenarios are investigated and tasks schedulability is formally proved before deploying them in electric vehicles. The proposed driver-assistance system offers many advantages. It decreases the risk of accidents and monitors the safety of driving. If, at some point, the risk index is above a certain threshold, an automated control algorithm is triggered to reduce it by activating different actuators. At the same time, it is lightweight and does not require any dedicated hardware, which in turn has a significant advantage in terms of battery life. Results show that the proposed system not only is accurate but also has a very negligible effect on energy consumption and battery life.

## 1. Introduction

Internet of Things (IoT) is a well-known paradigm of ICT (Information and Communication Technologies) and is attributed to the advanced connectivity of several elements, such as systems, devices, and services [1,2]. IoT has been evolving significantly since its inception. The earlier IoT focused on object connectivity, while the modern IoT focuses on real-time object interaction, creating a new generic term for the Internet of Things in real-time (RT-IoT) [3,4]. One of the enabling technologies of RT-IoT is the introduction of automation in vehicles, often known as driver-assistance systems. The benefits of an Electric Vehicle (EV) include the lower running costs and protection of the environment from the gases emitted from gasoline engines. However, modern studies have been focusing on making EVs comfortable with autonomous features. Some autonomous features such as self-parking and crash avoidance have already been in practice [5,6]. The development and research on EVs have not stopped, but the focus is significantly shifted towards autonomy in vehicles.

Driver-assistance systems possess a true potential to improve road safety and to decrease the risk of crashes as many of such crashes are caused by driver errors such as alcohol, drug, or fatigue [7,8,9]. There are a variety of driver-assistance systems such as adaptive headlights [10], anti-lock braking system [11], drowsiness monitoring [12], and Light Detection and Ranging (LIDAR), to name a few. However, these systems are costly, and for each system, a certain electronic control unit (ECU) must be installed on the vehicle, which puts an extra load on the battery of the vehicle. It is a massive challenge considering the importance of battery performance in electric vehicles. Consequently, there is still a gap in the literature to address such a crucial issue.

In this paper, the concept of a driver-assistance system is approached as a combination of real-time IoT tasks. These tasks are executed on edge nodes, and consequently, various components are offloaded from EVs. Edge nodes send and receive commands to/from EVs. This approach is better in terms of cost and flexibility. The system can be implemented using relatively cheaper hardware such as Rasberry Pi. An IoT server is deployed on Raspberry Pi sending and receiving commands to the vehicle in real-time and, thus, plays a vital role in reducing the overall cost. Moreover, it can also mitigate the need to install multiple dedicated ECUs for their respective operations, such as anti-braking and drowsiness detection.

Nevertheless, implementing the assistance system in EVs using the proposed approach faces several challenges. First and foremost, the guarantee to execute the task within an IoT network has been an enduring hurdle and cannot be 100% assured due to the nondeterministic nature of network delays. In some recent research studies, however, this can be estimated [3,13], paving a way to redesign the system using this approach. Secondly, since EVs perform certain tasks simultaneously at a specific interval of time, proper attention should be made to ensure the correct execution of them. Finally, EVs are suffering from a power minimization problem. Battery optimization has also been a critical issue; therefore, a system should be designed in such a way that it should not affect the performance of the battery with this additional processing. Nonetheless, implementing it within the road network has the potential to significantly reduce the number of crashes caused by drivers through the gradual removal of human interventions [14].

A typical driver-assistance system within an EV is thought to be an instance of a mixed-criticality system (MCS) of real-time tasks. The primary challenge is thus the assurance of real-time tasks with different criticality in such a way that the minimum number of tasks dropped and priority is given to highly urgent tasks. For instance, the system will always give more priority to a task which, if missed, leads to an accident than a routine task which has no potential associated hazards. One of the contributions of this paper is to investigate the primary drivers of crashes of EVs using safety scenarios, such as rainfall, light intensity, and surface friction. A driver-assistance system is designed to compute the accident-risk index (*ARI*) based on sensor data. If the *ARI* value is above a predefined threshold, the EV is declared to be in an unsafe driving state. In the end, control tasks are scheduled using a simple control scheduling mechanism to mitigate the risk of accidents. A task management tool is implemented to simulate these control tasks and to formally determine the schedulability of the control tasks. The proposed driver-assistance system is implemented on Raspberry Pi-based edge nodes to offload some of the processing from EVs. Consequently, it increases the overall battery time, which is vital for the design of EVs. Experiments are carried out to assess the reliability of the proposed assistance system with tradition real-time preemptive scheduling algorithms such as Earliest Deadline First (EDF) [15] and Rate Monotonic (RM) [16]. As part of the proposed system, a control scheduling algorithm is also designed and is evaluated with a modern RT-IoT-based Fair Emergency First (FEF) [13] for complex and hybrid task sets. The comparative results indicate that the task-dropping rate of highly critical tasks is decidedly less in the control scheduling algorithm as compared to the other general scheduling algorithms. The proposed system impacts on power consumption are also conducted, and it has been found to have a little impact on the overall battery time.

The rest of the paper is organized as follows: Section 2 describes related works in EV and driver-assistance systems. Section 3 presents system modeling for the prototype. Section 4 describes task analysis modeling. Section 5 presents the mechanism to generate datasets. Section 6 overviews the tools and technologies of the driver-assistance system prototype and describes different performance metrics. Section 7 presents a detailed discussion based on the findings of the study. Section 8, finally, concludes the paper and identifies future directions.

## 2. Related Work

The advancement in RT-IoT has paved the way for automating the operation of a vehicle systematically. Traditionally, numerous efforts have been made to investigate the safety impacts of EVs based on a variety of different approaches. In Reference [17], the authors assumed the near-elimination of errors caused by humans. It is crucial in the sense that the main factors of around 90% of accidents in the United States are due to drivers’ mistakes. In another study [18], a mechanism to detect fatalities has been proposed, where the functions of an EV are mapped on five different layers of crash data such as location, precrash scenario, vehicle speed, road conditions, and driver state. An autonomous testing institute based on California state provides real-world data which have been utilized in recent studies [19,20,21,22]. Schoettle et al. [20], for instance, found that EVs were not the main culprits in an accident and that the severity of injuries was lower in cases where the crashes involved autonomous vehicles rather than human-driven vehicles (HVs).

In order to avoid crashes, one of the primary approaches is the introduction of an advanced driver-assistance system (ADAS). ADAS technologies are well in the market for different safety measures such as anti-braking systems, radar, and LIght Detection and Ranging (LIDAR), to name a few. These systems are becoming as sophisticated as the ratio of accidents in vehicles equipped with ADAS is decreasing significantly. However, they use a system-on-chip approach and use a dedicated Electronic Control Unit (ECU), making it hard to modify and inflexible. Consequently, there is a need to have a consolidated application to perform similar actions as ADAS in a low-cost and flexible way [23,24]. The main difference is that, if modern technologies such as edge computing and RT-IoT are considered in the design of EVs, they can help in offloading the system-on-chip ECUs. It has been suggested in these studies that it is quite possible to model an autonomous EV as an RT-IoT system [13,24]. It is envisioned that modern EVs are real-time IoT systems with the constraint of energy and battery. Thus, task management and scheduling remain fundamental and must be formally proven before deploying it in the physical domain.

Driver-assistance systems are commonly employed to improve road safety and to avoid crashes. In a global status report on road safety [25], particular scenarios are highlighted, which must be catered to ensure safety. All of the scenarios were regarding the environmental condition, driver condition, and vehicle condition. For instance, road status, weather conditions and traffic conditions, driver status such as drowsiness and being drunk, and assurance of right speed and vehicle conditions are among the leading legislative actions. In another report [26] based on UK safety data, the same parameters were also streamlined as the topmost factors affecting road safety. It was categorized as driver competences, travel conditions, vehicle capabilities, and policy options arising, which inherently focuses on the aforementioned scenarios. Lynn et al. [27] suggested that road surface conditions, weather conditions, and lighting conditions are among the leading factors affecting road safety.

Apart from sensing tasks, control algorithms should also be carefully selected in the design of driver-assistance systems. The selection of a control algorithm or control system synthesis is not possible without a mathematical model of complete actuator structure [28,29]. Mathematical models can be used in the process of implementation of control algorithms (control based on the mathematical model) or in simulation—robust control algorithm checkups, in which implementation does not require familiarity with a mathematical model. When selecting a control algorithm, one of the first criteria is whether the management would require that the control ensures the system’s achievement of desired discrete values of position or force (setpoint mode) or whether it will have to ensure that the actual value of the output reflects the change of the desired input value. It is evident that, when it comes to the rocket movement, the electro-hydraulic system is required to have good tracking features because the desired value is continuously changing over time [28]. In EVs, the role of control algorithms can include functions like braking strategies [30,31], improved manoeuvrability [32], rollover prevention [32,33], and braking control [34] to avoid wheel lock [35].

EVs are equipped with sensors which sense ambient scenario, and the contextual data is processed in a central staging place, commonly the cloud, to find some unwanted patterns. These patterns trigger specific tasks that can be performed by actuators. The use of rule engines such as Drools to intelligently predict some actions based on the sensing data is being witnessed in modern research studies on autonomous EVs [24]. The tasks, if ensured to guarantee their deadline, can assist drivers without the need to deploy expensive ADAS hardware. Nevertheless, the tasks performed in a typical driver-assistance system are of two types; sensing tasks and control tasks. Either way, they need to be monitored for correct execution, and the schedulability of mixed-criticality task must be formally proved. In this regard, the first research on mixed-criticality scheduling on the multiprocessor platform was presented in 2009 and used slack shifting to schedule different criticality tasks [36,37]. Similar efforts such as Linux Testbed for Microprocessor Scheduling in Real-time Systems (LITMUS-RT) [38] and Bommert [39] were proposed for proper load distribution in mixed-criticality multiprocessor systems.

Kritikakou et al. [40] provided a new architecture, in which tasks are distinguished as only two levels: HI-criticality and LO-criticality. The idea is later backed by S. Baruah et al. and Zhang et al. [41,42]. Other scheduling algorithms include hierarchical scheduler [43,44,45] based on Scheduling Framework For Fast Prototyping (SF3P) [46] and is used to explore and prototype a hierarchical composition of the real-time scheduler. A younger algorithm for emergency first scenarios of complex tasks having different types was proposed by Malik et al. [13] in IoT space and suggested that, if tasks are carefully modeled, it will never drop a hard-deadline task. The current position of the state-of-the-art is summarised in Table 1.

## 3. System Model for Task Modelling in Proposed Driver-Assistance System

Driver-assistance technologies are focusing more on the automation of basic driving tasks. These tasks, as said earlier, are either control tasks or sensing tasks. In general terms, most of the sensing tasks are periodic tasks, while most of the control tasks are event-driven tasks. In this section, we will cover the basic methodology of task modeling as a fundamental step for the implementation of the proposed driver-assistance system concerning the requirements of RT-IoT systems in general and EVs in specific. Since EVs perform real-time tasks with mixed criticality, the fundamental component of the proposed system is a task. A task τ can be characterised by certain parameters such as the start time $s_{i}$, release time $r_{i}$, execution time $c_{i}$, and ending time $e_{i}$. Algorithms’ parameters such as worst-case execution time (WCET) $C_{i}$, period $T_{i}$, deadline $d_{i}$ and relative deadline $D_{i}$, core index $N_{i}$, and criticality $X_{i}$ are statically assigned. A task’s parameters, along with their description, are summarised in Table 2.

**Figure 1:**
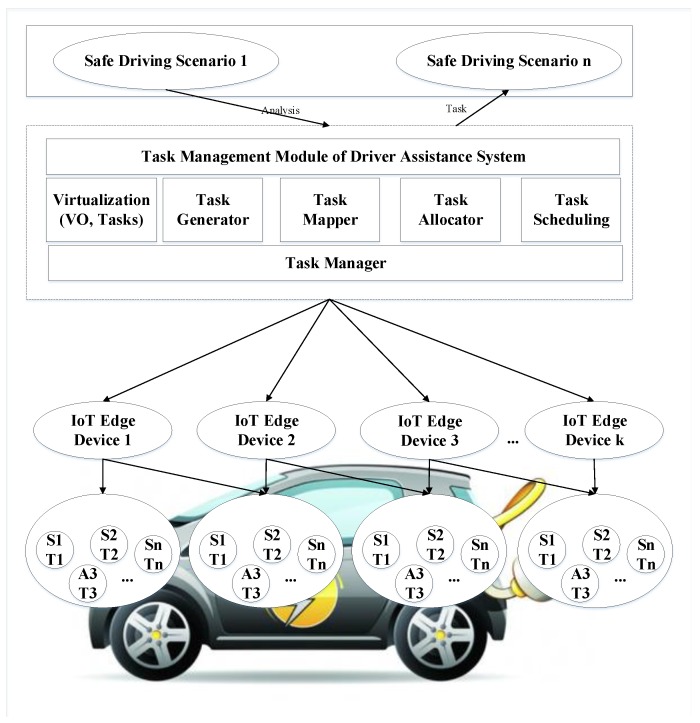
General system model of task modeling in driver-assistance system.

Scheduling and optimal allocation of resources in a typical EV is a multi-objective optimization problem. In this case, the objective is to minimize the dropping rate and battery consumption and to maximize the onboard CPU utilization. Figure 1 exhibits the general system model of task modeling and analysis in the proposed driver-assistance system.

There are different scenarios that can be considered in modeling the safety of EVs. Input tasks are generated based on these scenarios. The task generator module is responsible for task generation. Afterward, the task mapper maps these tasks on virtual objects (VO). A VO is the in-system representation of its corresponding physical sensor or actuator and is realized by the virtualizer module. The task scheduler schedules these tasks based on their priority, and finally, the task allocator module deploys them on physical devices.

### 3.1. Accident Risk Index Formulation

To model a safe EV, a certain degree of certainty must be ensured. Accident Risk Index (*ARI*) characterizes the monitoring status of tasks in execution and the probability of tasks dropping highly critical tasks and low critical tasks.

Suppose a *w* vector represents the impact weights and *S* represents the vector showing the corresponding sensors readings for all scenarios. The impact weights are set based on the severity of the potential accident. A higher impact weight is assigned if the risk associated with the accident is fatal. The weights are assigned based on the priority of factors contributing to road safety, which have been highlighted in many literature studies [47,48,49]. At this point, the weight is statically assigned, which best reflects the priority of the vehicle scenario. However, in recent studies, weights can be dynamically assigned by employing artificial intelligence techniques such as neural networks [50]. Similarly, the weights can also be dynamically adapted using metaheuristics algorithms such as Genetic algorithm [51]. However, it is not the focus of this work as the goal is to design a lightweight solution that works based on the predefined context by experts.

The ARI is defined as the vector multiplication of weight impact ω and sensor reading impact *S* as shown in Equations (1) and (2).

(1) $$ARI=\omega S_{i}$$

The total ARI is the weighted sensor impact of each sensor reading, as shown below.

(2) $$ARI=\sum_{j=0}^{n-1}\omega_{j}S_{ij}$$

**Figure 2:**
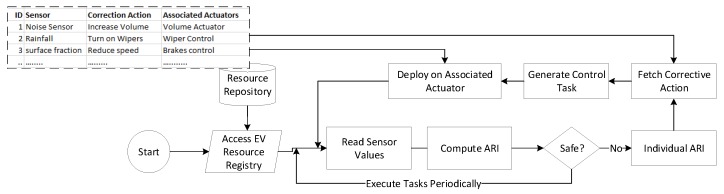
Flow diagram of EV safety profiling and corrective actions in a driver-assistance system.

Equation (2) dictates that the ARI of each scenario contributes to the total ARI value and, hence, to the safety state of the vehicle. As described earlier, there are numerous sensors installed in EV sensing ambient scenarios, and for each sensor, corrective action is defined in case the sensor reads a value which leads to an unsafe state. For this, actuators are deployed to take control and to aid in maintaining road safety. For instance, if blurriness is detected, the corrective action would be turning on the fan to remove the fog, and similarly, if the light condition is low and the road is wet in case of heavy rainfall, the corrective action would be the reduction of speed and turning on headlights. Nonetheless, when the sensor values lead to an unsafe ARI, the corrective actions use the actuator to control it and bring it back to the normal state. Every sensor value is normalized between 0 and 1, indicating a minimum risk index and maximum risk index, respectively. For instance, for a surface friction sensor, the value of the coefficient μ is in the range of 0.7 to 0.4, with 0.7 indicating normal road condition while 0.4 indicates extremely wet roads [52]. Therefore, the sensor impact $S_{i}$ is the impact of the sensor reading towards the ARI. The general formula for $S_{i}$ is shown by Equation (3).

(3) $$S_{sfi}=\left(f\left(max\right)-f\left(min\right)\right)\frac{\left(S_{v}-S_{min}\right)}{S_{max}-S_{min}}+f\left(min\right)$$

where f(max) and f(min) are the normalisation functions which are set according the scenario. For friction, if the sensor value is maximum, i.e., 0.7, the value we consider is 0, and if it is 0.4, the impact value is 1, which implies that this contributes the maximum to the ARI. The flow of computing *ARI* based on sensor values and controlling *ARI* based on sensors’ respective corrective actions are demonstrated in a flow diagram shown in Figure 2. As shown, the sensor values are read periodically, and based on them, the ARI is computed. If the ARI is high, individual *ARI*s are tracked down to find the culprit scenarios which lead the EV into an accident-prone state. As the value of individual *ARI* is normalized, the sensor with an *ARI* with more than a 0.5 value and high weight indicates the topmost culprits, and for this, the corrective actions are fetched from the repository and performed on the associated actuators. The sensor values are again read to determine if the value is safe or not, and this process continues.

### 3.2. Control Scheduling

It has been discussed in the earlier section that the value of ARI determines the safe driving. If the value of ARI is high, corrective actions are performed to bring it back to normal. Consequently, these control actions have more priority because they have more influence on the ARI, and consequently, they are scheduled first. That being said, the scheduler will check the control action priority, and based on the priority, it will schedule the correct action.

**Figure 3:**
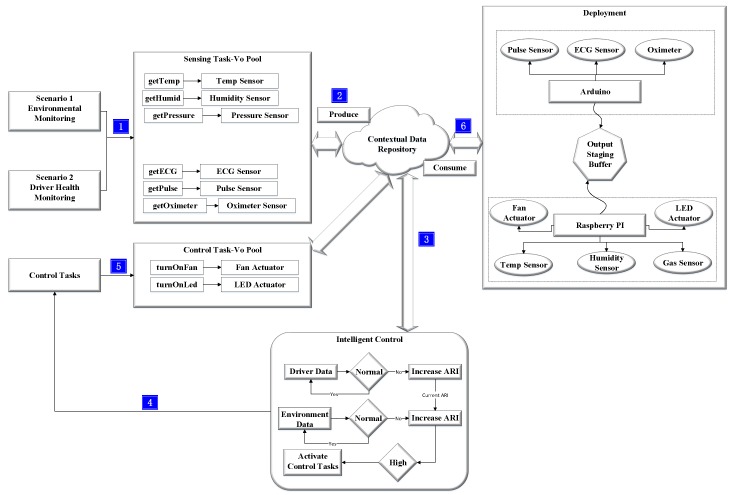
Control scheduling flow based on risk index profile in a driver-assistance system.

Figure 3 shows the flow of control scheduling. Data are taken from various sensors and processed in the staging area to find the risk index. If the risk index is high, it generates control tasks to avoid the possible risk.

## 4. Task Analysis and Modeling Based on Safe Driving Scenarios

In this Section, the analysis of possible scenarios are investigated and, based on them, tasks are generated. The tasks are summarised in Table 3. The tasks are categorized into three main categories: Input tasks, output tasks, and process tasks. Input tasks primarily collect environmental data using sensors. Output tasks control smart things based on the input data. Process tasks use some logical system to estimate the duty cycle of controllable things. For instance, the input task can be environment monitoring for rain detection, distance calculation, temperature sensing, and humidity sensing.

Process tasks assess input parameters to logically find the probability of some events and the duty cycle of controllable actuators. Finally, output tasks are mainly responsible for controlling actuators such as wipers and windows. In Table 3, the ID of the task determines the type of the task. For instance, task-i01 is an input task, task-o11 is an output task, and task-p15 is a process task. Every output task has a duty cycle, which determines the intensity of controllable things. If an actuator is targeted by more than a single task, the control scheduling algorithm fairly allocates the task to ensure safe driving and fair access. In the following subsections, we will derive different scenarios that directly affect the ARI.

### 4.1. Rainfall

Weather conditions account for different risks. Rainfall plays a crucial role in the sense that, in heavy rain, the visibility is poor and the roads are wet, which leads to a higher risk of accidents. Various sensors detect rainfall, as outlined in Reference [53]. According to the sensor in Reference [53], rainfall can be detected based on the sensor value range. Sensor-generated values between 0 and 300 indicate heavy rain, between 300 and 500 indicate mild rain, and no rain otherwise. The formula for $S_{i}$ is shown in Equation (4), which is a specialized version of Equation (3).

(4) $$S_{rfi}=\left(f\left(max\right)-f\left(min\right)\right)\frac{S_{v}}{500}+f\left(min\right)$$

where f(max) is the impact value taken when the value of the sensor is 500 or more and f(min) is the impact value taken when the sensor value is 0. The corrective actions taken include the reduction of speed in case of heavy rain, turning on the fan in case of fog, and turning on headlights in case of weak light intensity.

### 4.2. Noise Intensity

The noise can be computed using the sound intensity level. Intensity is defined to be the power per unit area carried by a wave. Power is the rate at which the wave transfers energy. Intensity *I* is given by Equation (5):

(5) I=P/A

where *P* is the power through an area *A* and the System International (SI) unit for *I* is W/m^2^. The intensity of a sound wave is related to its amplitude. For instance, the rustling of plants’ leaves are at 10 dB, and the sound intensity of a jet airplane is 140 dB.

The sensors for noise generate values in dB, and based on the reading, the volume of the radio is adjusted as a corrective action. The formula for $S_{i}$ is shown in Equation (6), which is the specialized version of Equation (3) and the standard formula of noise intensity:

(6) $$S_{nii}=\left(f\left(max\right)-f\left(min\right)\right)\frac{\left(P_{v}-60A\right)}{A(140-60)}+f\left(min\right)$$

where f(max) is the impact value taken when the value of the sensor is 140 dB or more and f(min) is the impact value taken when the sensor value is 60 dB or less.

### 4.3. Surface Friction

Friction is the force resisting the relative lateral motion of two solid surfaces in contact. Friction can be measured by the coefficient of friction μ, which is a dimensionless scalar describing the ratio of the force of friction between two bodies and the force pressing them together. μ depends on the material being used. For instance, ice on steel has a lower μ than rubber on the road. Under normal conditions, μ ranges from 0 to 1. For normal weather, the value of μ lies in the range close to 0.7, but for wet road, it falls to 0.4 [52]. The surface friction is crucial for safe driving because, if the road is wet, the surface friction will be lower than the normal road, and as a result, the road will not grip the tire when applying the brakes. Therefore, after applying brakes, the distance before finally making the car static will be slightly high. This will make the likelihood of hazards also high. Therefore, as a safe driving precautionary measures, we have to be aware of the road surface and the car needs to be aware of the likelihood of the hazards at every instant. The formula for $S_{i}$ for surface friction is shown in Equation (7):

(7) $$S_{nii}=\left(f\left(max\right)-f\left(min\right)\right)\frac{\left(P_{v}-0.7A\right)}{A(0.3-0.7)}+f\left(min\right)$$

where f(max) is the impact value taken when the value of the sensor is 0.3 or less and f(min) is the impact value used when the sensor value is 0.7 or higher. For decreasing the risk of hazards caused by surface friction, the vehicle speed should be reduced and a proper safe distance should be maintained as a corrective action.

### 4.4. Wind Speed

Wind speed is a crucial parameter and must be considered as an environmental parameter for the safety modeling of EVs. Wind speed can be measured in different units. Some of the units are Knots, Beaufort, m/s, km/s, and mph. Wind speed can determine the environment’s favorability to driving. In high intense wind, it is highly likely that a car can meet accidents due to air interference. The air interference of the car with wind makes the car speed abnormal, and the driver cannot control the car with normal force; therefore, wind speed can also be made into consideration. There is a different range of wind intensity for different scenarios. For instance, calm is considered as 0 Beaufort and 1 knot. It is a condition in which the wind speed is equivalent to 1.85 km/h. Similarly, light air is in the range of 1–3 knots, and the air is assumed to be light if it falls between 1–5 km/h. Different wind speed accounts for different *ARI*. A high value of wind speed induces high value of *ARI*, which would mean the EV is not in safe driving status.

### 4.5. Blurriness Detection

If the temperature of the internal environment of the EV and the outer environment is different and humid, the front glasses get blurred over time. It normally happens in overcast weather conditions. The blurriness can lead to poor vision of the driver and can increase the ARI as a result. Therefore, if an image is blurred beyond a certain value, some control actions such as turning on the fan and heater need to be necessitated to overcome the blurriness. The minimum the value is the maximum is the blurriness in an image [54].

### 4.6. Camera Images for Detecting Head Pose and Drowsiness

We can extract two kinds of information from the image captured from the camera installed in front of the driver. First, head pose [55] can be predicted with a series of Convolutional Neural Network (CNN) and by detecting the required angle. The angles are −45, 30, 15, 0, 15, 30, and 45. Secondly, the eye flick rate is recorded to predict whether the driver is drowsy or not [56]. Eye flick rate is determined using basic image processing to find the Eye Aspect Ratio (EAR). If the eye is closed, the aspect ratio is low, whereas if the eye is open, the aspect ratio is high. If for some time the aspect ratio drops and does not rise, it anticipates that the user is drowsy and an alert will be resonated to the driver. The low value of EAR indicates the drowsiness of the driver, and at that instant, the notification will be popped out.

### 4.7. Brake Status

Brake status is defined as the relation between distance per unit power of brake, often known as the braking distance and the fluid level. Braking distance depends on the slope of the road, the speed of the car, and the surface friction of the road. If the external parameters, such as surface friction and slope of the road are taken constant, then the brake status coefficient δ, indicating the effectiveness of brake, is given by the following equation:

(8) $$\delta =s\frac{F}{D}$$

where *s* is a constant indicating external factors such as the speed of the vehicle and slope of the road, *F* is the fluid level, and *D* is the distance per unit power of brake. Fluid level is fundamental since, if the fluid level is too low or the fluid contains too much water, the brakes will not work as expected. A brake sensor finds the level of fluid and the viscosity of the fluid to determine the status of the brake, and the warning signals are shown in case of undesired values. Thus, high fluid level and less distance upon applying brakes advocate the soundness of brakes of an EV.

### 4.8. Tire Status

The occurrence of road safety is highly correlated with the status of tires. A report on the German In-Depth Accident Study (GIDAS) suggested that the database has a significant entry in which accidents are caused by the poor tire status, and thus, tire aging and pressure monitoring sensors must be instated to predict such hazards in advance. Tire status is defined as a function of distance per brake and pressure of air in the tire. It also depends on weather conditions, particularly temperature. The tire status coefficient δ is a term indicating the condition of the tire and is given by the following equation:

(9) $$\delta =r\frac{P}{D}$$

where *r* is constant and indicates the road condition, *P* is the air pressure in the tire, and *D* is the distance per unit power of brake. We generated data using the equation, and 5000 entries are created based on the above equation, which will be described in subsequent sections.

### 4.9. Car Distance

The distance between cars is another essential factor defining the likelihood of fatality. If a car has high speed and, at the same time, has little distance from other cars, the chance of an accident is high. Various studies have suggested that a safe distance among cars must be ensured. It has been indicated that the difference in speeds among cars is the most important factor in finding the risk of crashes. In either case, a higher speed of the car would mean more braking distance and, hence, more chance of accidents. Thus, the risk of accidents is directly proportional to the speed of the car and to the positive difference of the speed of the leading car, and it is inversely proportional to the distance between the cars. The safe distance is the sum of the distance covered during the reaction time of the driver and the braking distance, which is given in Equation (10).

(10) $$D=D_{r}+D_{b}$$

where $D_{r}$ and $D_{b}$ are the reaction distance and braking distance probabilities. According to the basic physics rules, the final velocity $V_{f}$ is given by the following equation:

(11) $$V_{f}^{2}=V_{o}^{2}-2ad$$

where −a represents the deceleration of the vehicle if final velocity $V_{f}$ is taken as zero, the distance *d* is given by the following:

(12) $$d=\frac{V_{o}^{2}}{2ad}$$

Substituting the values in Equation (10), we get the following:

(13) $$D=t_{r}V_{o}+\frac{V_{o}^{2}}{2ad}$$

## 5. Tasks Dataset Generation

**Figure 4:**
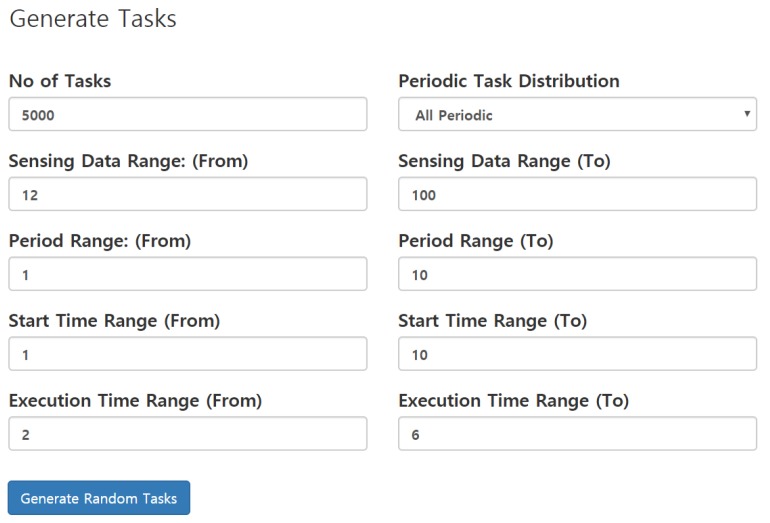
Sensor task creation generation interface.

In this section, a detailed mechanism of the dataset generation is elaborated. The data are generated with a pseudo-random mechanism in such a way to resemble physical sensor readings. The mere random generation is not practical since some of the values are not possible if deployed in real scenarios. For instance, if just a random generation is made, the rain value in one second may dictate heavy rain, while in the next second, it may dictate “no-rain”. Therefore, we made the data generation pseudo-random in which the next data point can be randomly generated within the range of the current data point. To make it further obvious, for instance, the current data point for rain is 300 and the range is ±5, and then, the following data point will be generated within the range 300−5,300+5. A task can be generated with a defined number of parameters. A sample sensor data generation form is shown in Figure 4. The data has been validated with a set of validation rules, as shown in Figure 4. The data can be either all periodic, percentage of periodic, or all event-driven. Once the form is submitted, the data is generated with different sampling periods, as explained in previous sections.

**Figure 5:**
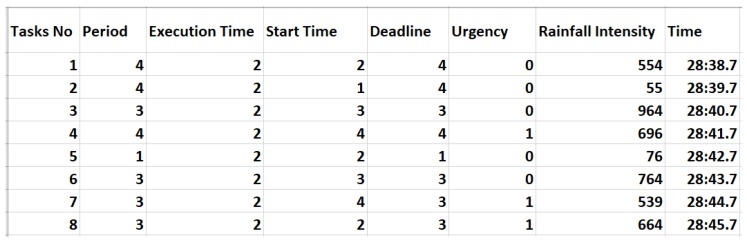
Generated datasets of rainfall tasks for a sampling interval of 1 s.

Once the form is submitted, the data is persisted in a Comma-Separated Values CSV file. We have created tasks for all the scenarios mentioned in previous sections. The data from a sensor can be taken at different time intervals. We consider five sampling intervals, and for each sampling interval, 5000 records are generated. For every scenario, the code to generate data is made a little different to reflect the requirements. We generate a dataset from sensors with different sampling periods. We consider 10 s, 5 s, 1 s, 0.5 s, and 0.1 s as sampling intervals. An example of sensor data for 1 s is shown in Figure 5. The sensor reading is smooth due to the pseudo-random generation and resembles real situations.

Similarly, datasets for other scenarios like camera images, blurriness detection, head pose angle detection, and the distance between cars are also generated in the same way. In the case of a camera image, the different angles show head pose. For instance, 45 and −45 angles show that the user is looking left and right, and thus, the risk of an accident is higher. For brake data, the data is collected in a tuple delimited by “-”, which shows the distance between the car and the fluid level. For the tire status scenario, the sensor data take care of the pressure of the tire and the distance covered per unit brake. For drowsiness detection, the EAR value is generated, which indicates different states of the driver.

Similarly, datasets for the camera image, car distance, tire status, and brake status are generated using the same approach. All the datasets have 5000 instances taken at sampling rates of 10 s, 5 s, 1 s, 0.5 s, and 0.1 s.

## 6. Driver-Assistance System Prototype Implementation

In this section, we overview the implementation stack of the proposed work and highlight the execution interfaces of different modules. We implemented the tool in Python as a core programming language. An overview of the tools and technologies used is in the implementation is presented and discussed in subsequent paragraphs. The EV emulator is developed on PC and deployed on Raspberry Pi for experimental purposes. The implementation environment is summarized in Table 4.

### 6.1. Control Scheduling Algorithm and Deployment of Complex Tasksets

The role of the scheduler is to execute the tasks fairly and to make sure none of the high priority tasks is missed. As covered in the earlier sections, the scenarios considered in this work are the ones that have more impact on the safety of the EV. However, even among these scenarios, some of them are more crucial than others. For instance, light intensity and rainfall are more crucial than noise intensity because low light and heavy rainfall can increase the potential risk of accidents, and hence, tasks of these scenarios need more consideration than the latter case. Nevertheless, the tasks are categorized into different classes based on the severity of the scenario. Table 5 summarizes the task priority and the reason why they are given the designated class.

**Figure 6:**
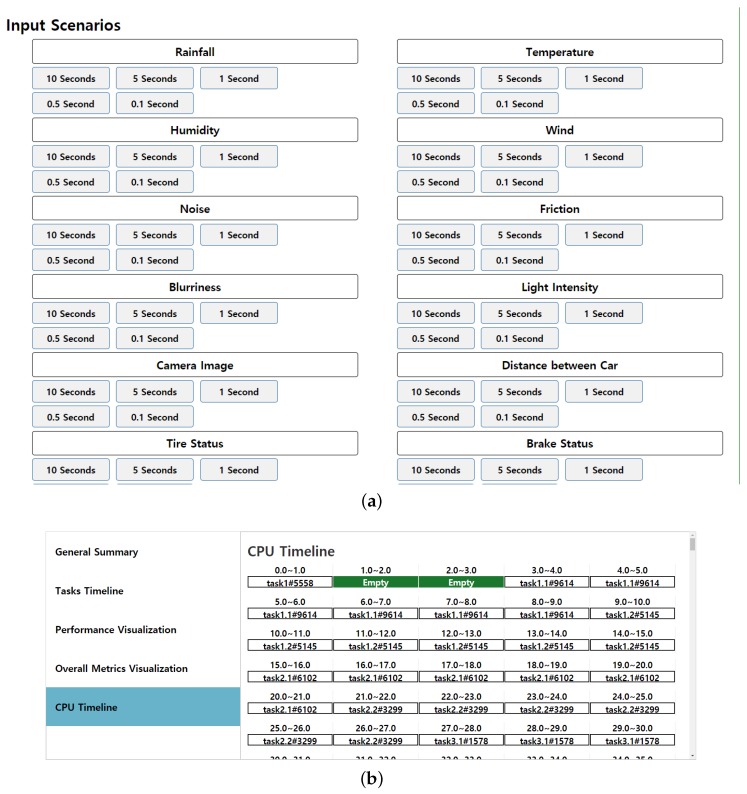
Interface for (**a**) task simulation scenarios and (**b**) scheduler interface.

The scheduling algorithm reads the sensors’ tasks, and based on the output of the sensors’ values, it generates output action priority. Figure 6 shows the interface of the scheduler. The scheduler reads task datasets of the derived scenarios and gives the dataset as an input to a priority-based scheduling policy. The scheduling policy schedules the tasks and reflects the scheduling footprints. Figure 6a shows the commands to activate a particular scenario. For each scenario, different sampling intervals are listed. The proposed tool can select a scenario and read the data of a specified interval, and once the data is read, it then prioritizes the output tasks.

In Figure 6b, the data is provided to the scheduler and the resultant CPU timeline is shown. Various color codes represent different categories of tasks. For instance, Task1 with pink color is a normal periodic task which is received at time 0. At this particular moment, no other high priority task has arrived yet, so it gets the CPU momentarily For the next couple of cycles, the CPU is free, which is indicated by a dark green color. Clock cycles 3 to 9 are assigned to event-driven task1.1, and this goes on until the hyper period amount of time. After this, the same pattern gets executed.

**Figure 7:**
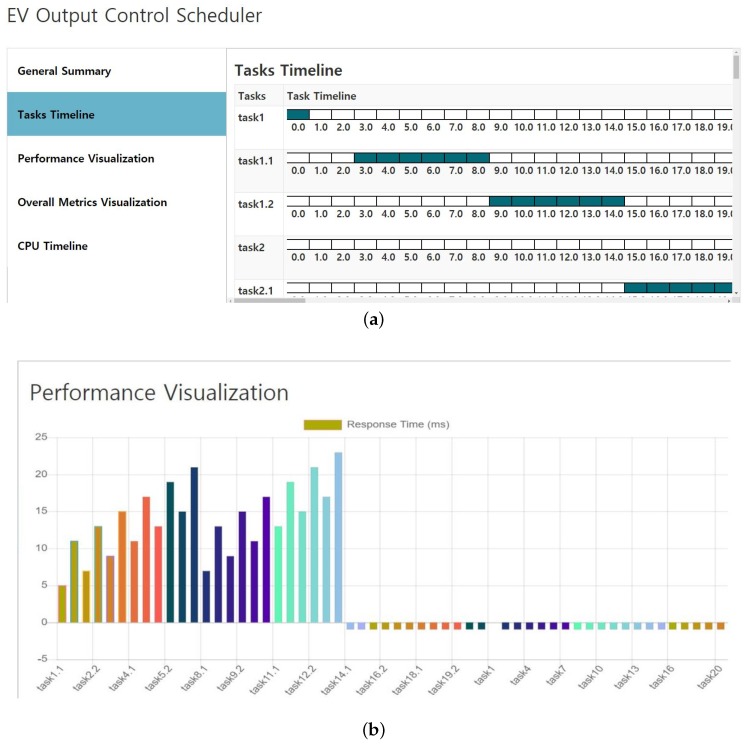
Interface for (**a**) task timeline and (**b**) performance visualization of EV output control scheduler.

Another interface is for the task timeline, which shows the individual tasks and its CPU occupancy status. For every task, the CPU time frame is shown along with the information on whether the task is using the CPU or not. The box on the task timeline represents 1 ms. The occupied CPU is shown in blue, while the empty one is shown in white, as shown in Figure 7a. It shows that the first task1 is assigned for one ms. After that, no incomplete task is left, so for the next couple of ms, the CPU is empty. At this point, task1.1 has arrived and is scheduled on the CPU. Task timeline is an essential visual component for any algorithm because it shows the status of every task. From the task timeline, one can easily spot the CPU occupancy and fairness of the scheduling algorithms. The continuous strips from top to bottom depict incoming tasks and their respective CPU clock cycles. Another significant aspect of visualizing is the response time chart and other metrics in a bar or line graph. We use Chart.js library based on the native JavaScript programming language. Chart.js provides the ability to visualize the CSV data in a graphical form to find a pattern that can be hardly spotted if looked at in a CSV form. For the control scheduling algorithm, the response time is represented in Figure 7b, which shows the task-wise response time after running the control scheduling algorithm. The response time is in the range of milliseconds. For some tasks, the response time is −1, which indicates that they were dropped and did not get the CPU. The response time 0 indicates that the task got the CPU the moment it arrived. Distinct color codes are assigned for different tasks for identification purposes and visualization. For this, Palette, a Python-based library, has been used. Palette is responsible for color manipulation. Color addition, Red Green Blue (RGB), Red Green Blue Aplha (RGBA), and every vector of color can be manipulated with ease.

### 6.2. Embedded System for Driver-Assistance System Prototype

In this section, we discuss deploying the system on embedded hardware. For this purpose, we use two Raspberry Pi devices acting as edge nodes on which IoT servers are installed and operated with the associated physical resources. The Raspberry Pi system is equipped with the necessary libraries and programming languages on top of Raspbian OS. On Raspberry Pi hardware, Raspbian OS is installed. On a fresh installation, the Java core programming language and Python 3.5 is installed. Some dependent libraries like Chart.js, CSV Parser, and CSV parser need to be available. Similarly, for listening to requests and for interacting with IoT resources such as sensors and actuators, a server is necessary. For this purpose, we use Flask. The technology stack is summarised in Table 4.

**Figure 8:**
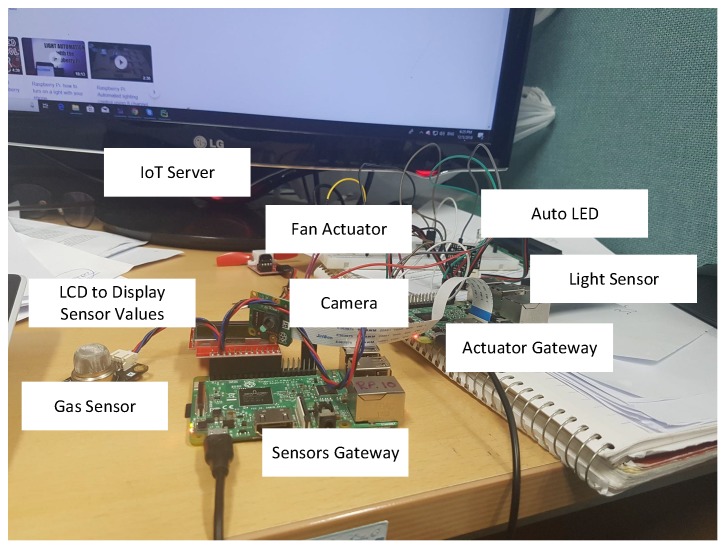
Embedded system for driver-assistance system prototype.

Raspberry Pi device, which acts as a gateway to the emulator application, has numerous resources attached. The resources are Barometric Pressure (BMP) sensor and a camera sensor. Moreover, it also has a fan actuator, which we use for wiper control, and LED, which we use for light control. Similarly, we use a speaker actuator for controlling the volume of the radio. We use two Raspberry Pi gateways; the first one communicates with actuators, while the second one communicates with sensors. The sensors’ gateway reads the sensor values, calculates ARI, and infers the control tasks. The scheduler prioritizes the control tasks and sends them to the gateway having actuators attached. The implementation environment is shown in Figure 8.

### 6.3. Performance Testing

**Figure 9:**
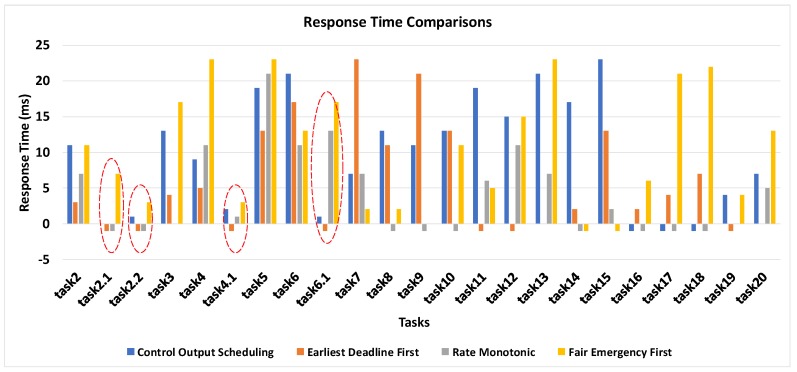
Response time comparison of the rate monotonic, earliest deadline first, fair emergency first, and control output scheduling algorithms.

In this section, we investigate the performance of the control output scheduling algorithm with classical EDF and RM algorithms and with a modern FEF algorithm designed for an IoT-based complex task set. EDF and RM are general-purpose preemptive algorithms based on the rule of static and dynamic priority. RM and EDF are iterative scheduling algorithms, which are based on the careful selection of the period of the task set. FEF is based on IoT-based approximation of hard real-time systems for complex task sets having different categories of tasks. Figure 9 shows the bar graph demonstrating the response time comparisons of the first 20 tasks. The task set is a mixed-criticality task of event-driven and periodic nature. The control tasks generated as a result of the occurrence of certain events are numbered in decimal points such as task2.1, whereas the IDs of periodic tasks are numeric, such as task1. The task set is supplied as an input load to the aforementioned scheduling algorithms. Two tasks are generated for each scenario, and in some scenarios, control tasks are also triggered, making the task set an ideal candidate to assess the performance. The response time of EDF and RM algorithms is not good for a mixed set of tasks containing both periodic and event-driven tasks. In both cases, the event-driven tasks are ignored due to the period values, which are 0 for event-driven tasks. However, for periodic tasks, the average response time is approximately 10 ms, which is not bad since, in IoT, the sensor tasks’ relevancy is normally in the order of 20 ms to 30 ms. In the case of FEF, the response time is on the lower end, and due to the dynamic support of complex task set nature, the urgent tasks are given priority, making it better than the first two algorithms. The proposed algorithm is designed specifically for control tasks, and by taking a keen look at the graph, the response time of all control tasks is close to zero, which means that the control tasks get executed whenever they are generated. The control tasks are highlighted in the graph to put more emphasis on the performance of the control output scheduling. For instance, if we look at the first control task with ID task1.1, the control output scheduling algorithm executes it with 0 response time and EDF and RM drops it, which is indicated by −1, while FEF executes it after a delay of 7 ms. FEF also gives priority to urgent tasks, but it considers urgent periodic and urgent control tasks of equal importance, and thus, the response time of control tasks is higher than the proposed algorithm.

### 6.4. Task Missing Rate

**Figure 10:**
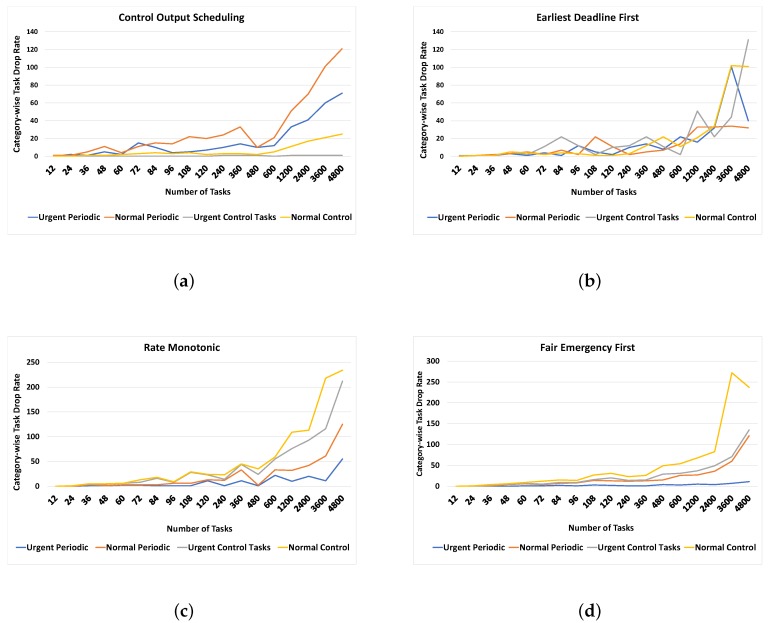
Effect of task dropping rate on increasing number of tasks: (**a**) control output scheduling, (**b**) earliest deadline first, (**c**) rate monotonic, and (**d**) fair emergency first.

In this section, the impact of increasing tasks and sampling rates on the abovementioned algorithms is investigated. Figure 10 shows the results demonstrated with a line graph. There are four categories of tasks as described before; urgent periodic, normal periodic, urgent control, and normal control tasks. The proposed control output scheduling algorithm mainly focuses on urgent control tasks, so irrespective of the increasing number of tasks, the task dropping rate of highly critical control tasks is always close to zero. For instance, the grey color indicates an urgent control task and has a very tiny percentage even for a considerable task set, i.e., more than 4000. In contrast, EDF and RM algorithms do not consider the type of tasks and make schedules based on other tasks’ parameters such as deadlines and periods. Therefore, the task drop rate is not distinguishable for different categories. In the case of FEF, the task dropping rate for urgent periodic and normal periodic are on the lower side, which means that the highly critical periodic tasks are given more priority at the cost of control tasks. FEF is the best candidate for a hybrid task set having mixed-criticality in the IoT environment. However, in this case, the purpose is a close-to-zero drop rate of control jobs, which is best suited in the case of the proposed algorithm. The limitation of this algorithm, however, is that it does not work as effectively and adaptively as FEF for a hybrid task set in which both periodic and event-driven tasks have equal importance.

**Figure 11:**
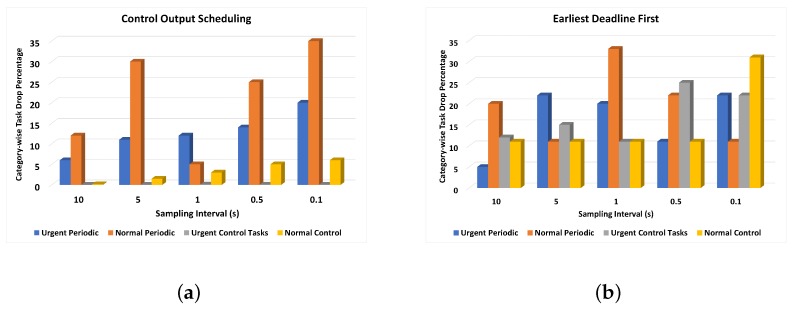

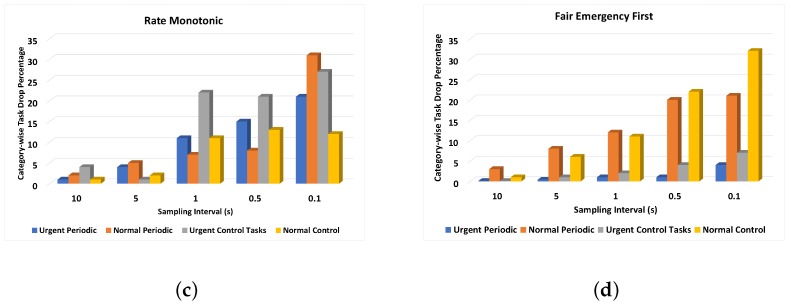
Task dropping rate impact on varying sampling rates using (**a**) control output scheduling, (**b**) earliest deadline first, (**c**) rate monotonic, and (**d**) fair emergency first.

The second case for which the task-missing rate is investigated is its impact on varying sampling interval. The data is taken from sensors at intervals of 10 s, 5 s, 1 s, 0.5 s, and 0.1 s as described in earlier sections. Figure 11 shows the bar graph showing the task dropping percentage for each algorithm for varying sample intervals and a fixed number of 1000 tasks. It is evident that the shorter the sampling interval, the faster the tasks will generate, and the scheduling will be more challenging as a consequence. In modern IoT applications, the sampling rate is on the higher side and the sensor reading tasks have more frequencies due to high-speed communication media and processing devices. Therefore, having more sensor reading tasks in unit time will have the chance to generate more control tasks. The bar graph shows that, for a sampling interval of 10 seconds, the task dropping percentage for each category is meager. In Figure 11a, the grey bar indicates that no urgent control task is dropped, which is the primary motivation of any assistance system for drivers. Although there is some notable number of dropped tasks, they are mostly normal periodic, but that too is 0.035% of total tasks. For EM and EDF, the task dropping percentage is balanced in all categories; however, with decreasing sampling interval, the task dropping percentage is increased. The average task drop percentage is higher than the proposed algorithm in both cases. In FEF, in contrast, the average task dropping rate is slightly better than the proposed algorithm, but over a keen look, it is evident that the task drop percentage of control tasks is much better in the case of the proposed control output scheduling algorithm.

### 6.5. ARI Analysis

**Figure 12:**
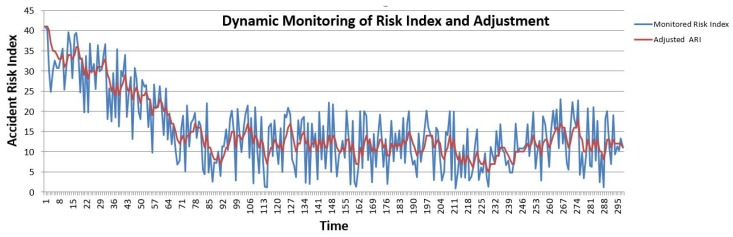
Effect of State of Charge (SoC) over time.

As described in previous sections, the ARI value must be adjusted with a certain range to reflect the safety of EV. Figure 12 illustrates that the *ARI* adjustment depends on the monitored sensor values. The blue line shows the computed ARI, and the red line is safe ARI, which is adjusted to make the EV in a safe driving state. The computed ARI oscillates, but the safe ARI is always the adjusted version of that sensor, which has the potential associated risk.

### 6.6. EV Battery Impact on the Proposed Driver-Assistance System

**Figure 13:**
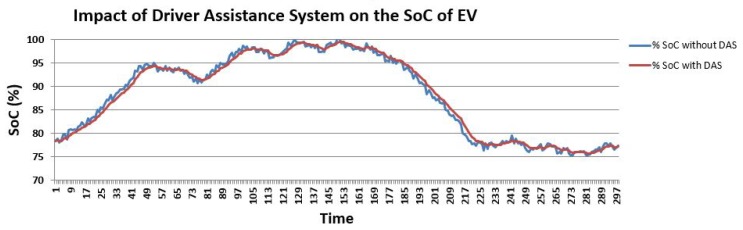
Risk index analysis and adjusted *ARI*.

As it has already been discussed that battery performance is one of the crucial attributes of EVs, a tool that has a drastic impact on the battery of EVs is of no use irrespective of its many uses. The proposed driver-assistance system is very lightweight as most of the processing work is performed on edge nodes, and the actuator installed only executes commands, which is a very minimal portion of the overall processing, and, thus, seemingly has no impact on the battery performance of the vehicle. Figure 13 depicts the percentage SoC of the EV in case the driver-assistance system is installed and in case it is not. The blue trajectory indicates the earlier case and the red trajectory indicates the latter case, which overlap most of the time. This indicates that, with the installation of the proposed driver-assistance system, it has a significantly low impact on the SoC. The *x*-axis represents the unit of time the EV is in the running state.

## 7. Discussion

In this paper, we have proposed the idea to redesign the driver-assistance system with a new approach to be generic as well as battery efficient. Traditional state-of-the-art driver-assistance systems such as Anti-Braking System (ABS) and LIDAR are well established due to their accurate results; however, they are designed for a dedicated purpose and, hence, cannot be utilized for other tasks. Moreover, these systems are deployed on ECU, which, in turn, are deployed on EVs. Consequently, they put an extra burden on the battery, which is highly undesirable. Keeping these hurdles in mind, we proposed the idea of a comprehensive system that performs many tasks to facilitate drivers and to avoid hazards by timely notifying drivers and by performing corrective actions. The proposed system is implemented using low-cost open-source hardware and IoT gateways acting as edge nodes. The edge nodes do all the processing and send commands to the vehicle, making it extremely power-efficient.

The dedicated scheduling algorithm always gives more priority to control tasks, which in most cases perform some corrective actions to avoid the risk of accidents. The results of the proposed system indicate that, even for a massive number of tasks, very few control tasks are missed. More importantly, the deployment of such a system will allow the offloading of dedicated hardware, which was affecting the performance of the battery at the cost of safety. This is witnessed from the battery performance of the system, which is slightly more than the vehicle with no driver-assistance system. Nonetheless, the proposed design can be a beneficial contribution to state-of-the-art. Nevertheless, the system is instead tested with real but simple scenarios, and thus, for a more complex scenario, the accuracy of these lightweight systems is worth investigating.

## 8. Conclusions

In this paper, we considered scenarios of EVs, which play a vital role in safe driving. We proposed a driver-assistance system that modeled tasks of sensing data and actuating data and based on contextual data from sensing tasks computed *ARI*, which determined driving state to be either safe or accident-prone. The generated datasets based on these scenarios were emulated in an embedded environment based on Raspberry Pi and a set of sensors and actuators. The tasks were fed into a control scheduling algorithm to assign priorities to output tasks based on the criticality and the impact on the value of ARI. From the experimental results, it has been claimed that no high-priority control task is dropped. The proposed system is compared with classical EDF and RM algorithms and modern FEF algorithms, and it has been found to have a better response time and that the task dropping percentage is very low for control tasks in comparison with other selected algorithms. In this study, we have considered test sensors and actuators and simulated data. A possible future direction of this work can be to deploy the proposed system on actual sensors and actuators installed in EV and to profile the value of the proposed tool in a physical EV testbed.

### 8.1. Implication for the Industry

This tool can be utilized in a very effective way in the industries which work in the automation of smart vehicles based on RT-IoT. It gives a unique perspective of EV to be a collection of real-time simultaneous tasks with a hard deadline. The proposed tool will enable industries to look down on the different tasks which drive a typical EV and investigates the critical tasks and noncritical tasks. Consequently, it can help in profiling the vehicle in software and to understand the processes inside a vehicle, which is of utmost importance given the critical nature of the possible consequences.

### 8.2. Implication for Academia

The proposed tool is based on the latest open-source tools and technologies and utilizes better design patterns such as Model View Controller (MVC), and it is intended to be open-source. Therefore, it can pave a great way for students and researchers to use it in research projects. Additionally, the conceptualization of a smart space and profiling it using a tool can aid a great deal in the overall comprehension of the domain knowledge. This tool will help as a great teaching tool to implement simple case studies of IoT smart spaces and can better understand the role of control scheduling and sensing scheduling of tasks in a standard IoT context.

## Figures and Tables

**Table 1:** Current position of state-of-the-art methods.

	Reference	Common Goals	Limitations
Road Safety and Risk Driving	[7,8,9,17]	Ensures road safety and avoids crashes and fatalities.	Not specific to electric vehicles and, hence, does not consider the battery and energy requirement.
Driver-Assistance Systems	[10,11,12]	Designs a tool to reduce the ratio of accidents by facilitating drivers and sending alarms and notifications.	Dedicated electronic control units (ECUs) for each specific function make the electric vehicle (EV) unnecessarily overloaded.
Automation Tools	[5,6]	Eliminates or reduces human intervention to reduce traffic crashes as the majority of the crashes are due to human such as fatigue and alcohol.	These tools are purely for automation inside any vehicle, and thus, they focus more on accuracy rather than on power efficiency.
Real-time Scheduling Algorithms	[15,16]	The traditional real-time algorithms mainly designed for an operating system have some defined goals, such as fair execution, CPU utilization, low response time, and maximum throughput.	These rather old solutions need to be tweaked a bit to adapt to modern applications. However, they are also considered as a baseline to test new algorithms.
Real-Time Internet-of-Things (IoT) Systems	[3,13]	Real-time system schedulers can be redefined with equal effectiveness in IoT systems considering 5G technology can ensure network delay within an upper bound. Scheduling algorithm fair emergency first (FEF) is proposed to execute a hard-deadline task.	There is any commercial tool which is based on this idea, making it a little unreliable for true hard real-time application.

**Table 2:** Task characterisation in mixed-criticality EV system.

Notation	Name	Description
$r_{i}$	Release Time of Task $\tau_{i}$	Release time is the time when the task is released. This parameter is used to describe the release time of a job of a task.
$c_{i}$	Execution time of $\tau_{i}$	Execution time is the processing time that a job takes.
$e_{i}$	Ending time of $\tau_{i}$	Ending time is the time when a job finishes its execution.
$d_{i}$	Deadline of task $\tau_{i}$	By the deadline, a task has to finish its execution. Whether a task has missed its deadline could be determined by comparing the deadline $d_{i}$ with the ending time $e_{i}$. It has usually the following relation: $d_{i}=r_{i}+D_{i}$, where $D_{i}$ is the relative deadline. If a task could not finish before its deadline, we call it a deadline miss.
$C_{i}$	Worst Case Execution Time	$C_{i}$ is the maximum length of time a task could take to execute on a specific hardware platform. Since it is very hard to get the real $C_{i}$ of a task, a measured maximal execution time is usually referred as worst-case execution time (WCET). Therefore, it is possible that a task exceeds its WCET; we call it overrun of task.
$T_{i}$	Period of periodic task $\tau_{i}$	$T_{i}$ is the execution period of task. In synchronous-periodic task set, T is referred to as the hyper-period of the task set.
$D_{i}$	Relative Deadline	Relative deadline is a predefined limit time scale, in which a task should have finished.
$N_{i}$	Core Index on a multi-core processor	The core index on the which the task is currently assigned.
$X_{i}$	Criticality of $\tau_{i}$	$X_{i}$ is the criticality level of a task. This parameter is crucial in the driver-assistance system.

**Table 3:** Input, process, and output task examples of EV for safe driving.

Task ID	Name	Description	Data	Source/ Destination
Task-i01	getTemperature	This task will get temperature according to the parameter	Temperature data	Temperature sensor
Task-i02	getHumidity	This task will get the humidity from the humidity sensor	Humidity data	Humidity sensor
Task-i03	getwindSpeed	This task will get the wind speed from the windspeed sensor	Wind data	WindSpeed sensor
Task-i04	getLightIntensity	This task will get the light intensity from the light sensor	Light data	Light sensor
Task-i05	getNoiseIntensity	This task will get the noise intensity from the noise sensor	Noise data	Noise sensor
Task-i06	getCamImageData	This task will capture the image of the front-using camera sensor	Captured image blob	Camera
Task-i07	getSurfaceFriction	Surface friction can detect how wet is the road and what is the safe speed to maintain a safe distance between cars	Surface friction data	Friction sensor
Task-i08	getFrontBlurness	In rain, the front and side glasses are blurred. This blurness can be removed by turning on the fan or by opening windows	Glasses images	Camera
Task-p14	compRainIntensity	This task will compute the rain intensity from the context data	Temperature, humidity, camera images, noise, blurness, surface friction	Rain speed, car speed
Task-p15	compNoiseIntensity	This task will compute the noise intensity from the context data	Noise sensor data	Radio volume
Task-p16	compBlurness	This task will compute the noise intensity from the context data	Glasses image data
Task-o09	controlWiper	This task will control wiper according to the parameter	Rain intensity, speed	Wiper actuator
Task-o10	controlVolume	This task will set the correct volume of the radio	Noise intensity data	Radio volume
Task-o11	controlWindow	This task will open or close the windows based on the rain intensity	Rain intensity	Car windows
Task-o12	controlSpeed	This task will control the speed of the car based on the rain intensity	Rain intensity, noise intensity	Accelerator, brakes
Task-o13	turnOnFan	This task will turn on fan to remove the fog from front glass	Camera data	Fan

**Table 4:** Implementation environment for EV emulator.

Component	Description
Hardware	Raspberry PI, PC
Operating System	Raspbian, Window 10
Memory	1 GB, 8 GB
Server	Flask Webserver
Libraries	Jinja, CSV generator, Bootstrap, Chart.js, Javascript, HTML and CSS
IDE	PyCharm and IDLE edit
Core Programming Language	Python 3.5

**Table 5:** Categorization of different tasks based on severity of scenarios.

Task ID	Class	Reason
Task-i01	Normal Periodic	As this task does not directly affect the risk associated with rainfall, it can contribute to finding the accuracy of rain.
Task-i02	Normal Periodic	Humidity can also help in finding the accurate prediction of rain because, in rainy weather, the humidity is high.
Task-i03	Normal Periodic	Normal wind speed has no associated risk with road safety, but heavy wind can lead to unsafe conditions.
Task-i04	High Priority Periodic	In poor light conditions, visibility is low and the risk of accidents is high.
Task-i05	Normal Priority Periodic	Noise is not directly related to the safety measures of the vehicle and road, but it can help the vehicle adapt to the environment by automatically adjusting the radio volume.
Task-i06	High Priority Periodic	Camera image contributes to collecting data, which are crucial in risk analysis, such as the drowsy state of the driver.
Task-i07	High Priority Periodic	If surface friction is low, the safe distance will also be low and, thus, needs to be adapted for safe driving.
Task-i08	High Priority Periodic	Blurriness leads to poor visibility and, hence, high risk of accidents.
Task-o09	High Urgency Event Driven	This is the highest priority task because failing to execute this task on time may lead to major accidents and loss of lives.
Task-o10	Normal Event Driven	The noise of the atmosphere can cause the radio volume to increase. It is a normal event-driven task. If it fails, it might not have as major a consequence as in the case of the wiper.
Task-o11	Normal Event Driven	This is also normally event-driven as windows do not contribute much to the safety of the car and people.
Task-o12	High Urgency Event Driven	The car speed under high intense rain can cause slipping, and brakes may not work; therefore, car speed needs to be lowered as a safety measure. Therefore, it is of high urgency in event-driven nature.
Task-o13	High Priority Event-Driven Task	Car fan should be turned on and off based on the blurriness of the front glass.
