# Erratum: Ahmad, S.; Malik, S.; Park, D.-H.; Kim, D. Design of Lightweight Driver-Assistance System for Safe Driving in Electric Vehicles

**DOI:** 10.3390/s19245380

**Published:** 2019-12-06

**Authors:** Shabir Ahmad, Sehrish Malik, Dong-Hwan Park, DoHyeun Kim

**Affiliations:** 1 Department of Computer Engineering, Jeju National University, Jeju 63243, Korea; shabir@jejunu.ac.kr (S.A.); serrym29@gmail.com (S.M.); 2 Electronics and Telecommunications Research Institute, Daejeon 34129, Korea; dhpark@etri.re.kr

The authors wish to make the following erratum to this paper [1].

Funding is incorrect and must be replaced by the following Funding:

**Funding:** This research was supported by the MSIT (Ministry of Science and ICT), Korea, under the ITRC (Information Technology Research Center) support program (IITP-2019-2014-1-00743) supervised by the IITP (Institute for Information & communications Technology Planning & Evaluation), and This work was supported by Institute for Information & Communications Technology Planning & Evaluation (IITP) grant funded by the Korea government (MSIT) (No. 2019-0-01456, AutoMaTa: Autonomous Management framework based on artificial intelligent Technology for adaptive and disposable IoT). Any correspondence related to this paper should be addressed to DoHyeun Kim.

The authors would like to apologize for any inconvenience caused to the readers by these changes.
